# Supplementary material for: A Glycolipid Glycosyltransferase with Broad Substrate Specificity from the Marine Bacterium “Candidatus Pelagibacter sp.” Strain HTCC7211
Source: Appl Environ Microbiol. 2021 Jun 25;87(14):e00326-21. doi: 10.1128/AEM.00326-21 (PMC8231724; doi:10.1128/AEM.00326-21)
Supplement: Supplemental file 1 — Table S1, Fig. S1 and S2. Download AEM00326-21_Supp_1_seq9.pdf, PDF file, 0.4 MB [file aem00326-21_supp_1_seq9.pdf]

Supplementary Table1 Determination of metals in GT_{cp}.

Sample analysed	Ca^{2+}	Mg^{2+}	Mn^{2+}	Zn^{2+}
GT_{cp} (μg/mg protein)	0.03±0.02	0.02±0.01	0.03±0.01	0.02±0.01
GT_{cp} (molar equivalent $\times 10^{-2}$)	2.75±0.03	2.98±0.02	2.09±0.02	1.18±0.01

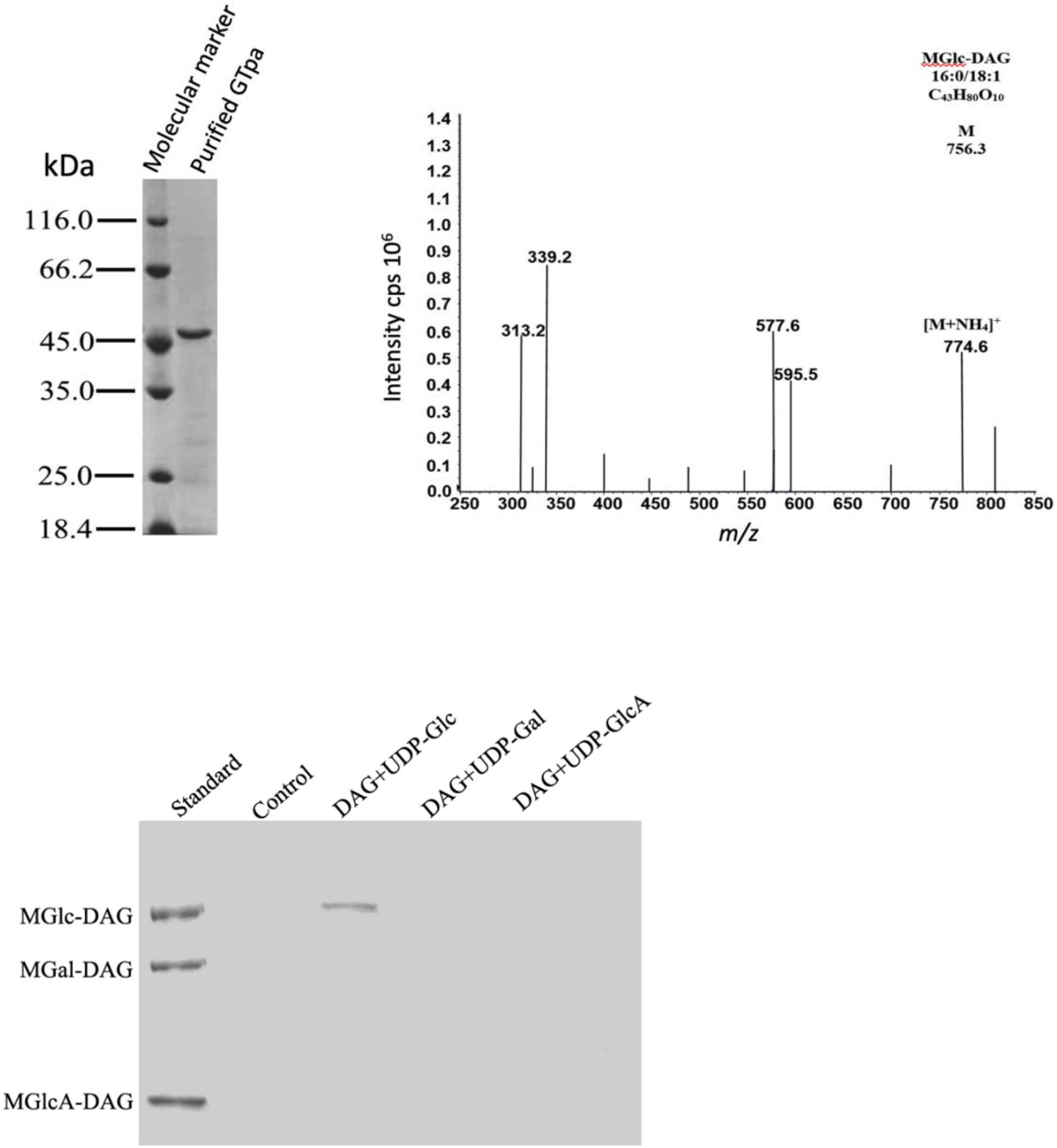

Figure S1 Top left panel, purified GT4 homology of *Pseudomonas* sp. PA14 (GT_{pa}). **Top right panel**, purified GT_{pa} only accepts UDP-glucose as the sugar donor and showed no activity towards UDP-glucuronic acid nor UDP-galactose (**bottom panel**). LC-MS of fragmentation spectra (top right) for MGlc-DAG were obtained from the products of the GT_{pa}-catalysed reaction. Thin layer chromatography (bottom panel) of the enzymatic reaction products with different UDP-sugar donors and diacylglycerol (DAG) as the substrate.

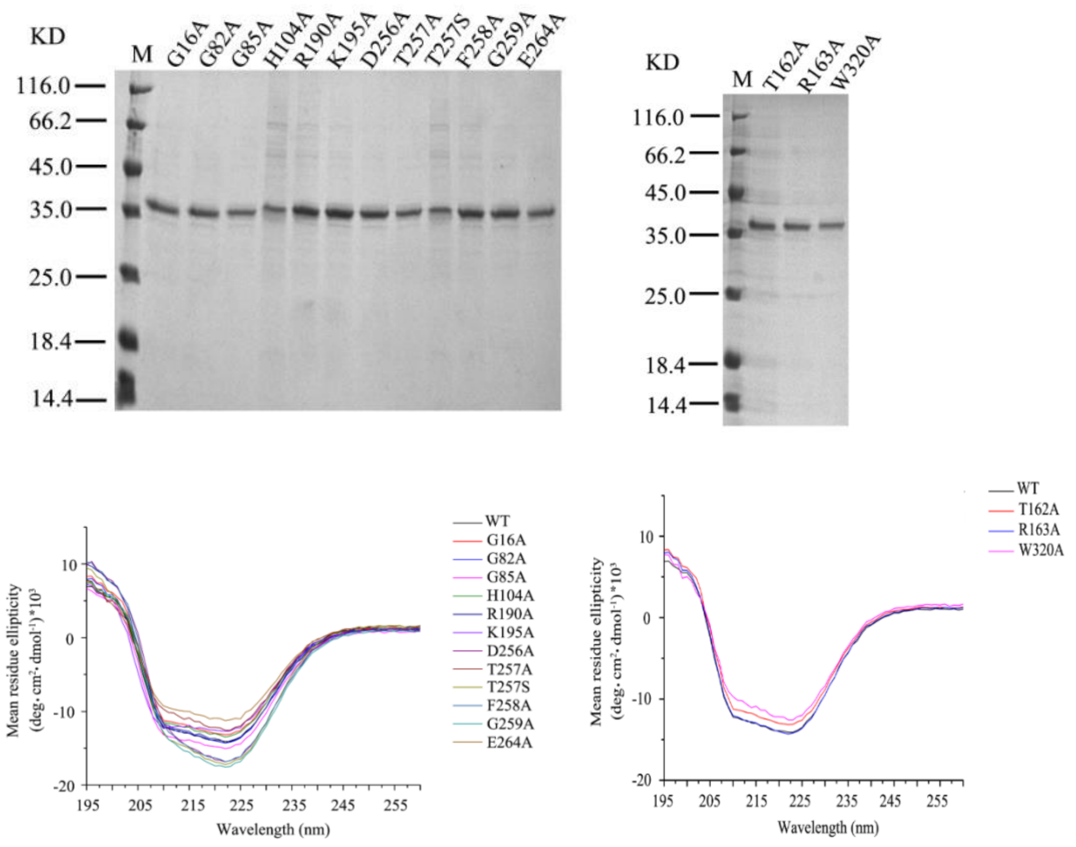

Figure S2 Top panels, SDS-PAGE of site-directed protein variants. **Bottom panels**, Far-UV CD spectra of wild-type GT_{cp} (WT) and site-directed protein variants.
